# Cross-sectional study of Facebook addiction in a sample of Nepalese population

**DOI:** 10.12688/f1000research.26923.2

**Published:** 2020-12-04

**Authors:** Alok Atreya, Samata Nepal, Prakash Thapa

**Affiliations:** 1 Lumbini Medical College, Palpa, 32500, Nepal; 2 Manipal College of Medical Sciences, Pokhara, Nepal

**Keywords:** Addiction, Crime, Internet, Nepal, Social Networking, Facebook

## Abstract

**Background: **Facebook addiction is said to occur when an individual spends an excessive amount of time on Facebook, disrupting one’s daily activities and social life. The present study aimed to find out the level of Facebook addiction in the Nepalese context and briefly discuss the issues associated with its unintended use.

**Methods: **A descriptive cross-sectional study was conducted in the Department of Forensic Medicine of Lumbini Medical College. The study instrument was the Bergen Facebook Addiction Scale typed into a Google Form and sent randomly to Facebook contacts of the authors. The responses were downloaded in a Microsoft Excel spreadsheet and analyzed using Statistical Package for Social Sciences version 16.

**Results: **The study consisted of 103 Nepalese participants, of which 54 (52.42%) were males and 49 females (47.58%). There were 11 participants (10.68%) who had more than one Facebook account. It was observed that 8.73% (n=9) to 39.80% (n=41) were addicted to Facebook.

**Conclusion: **When used properly Facebook has its own advantages. Excessive use is linked with health hazards including addiction and dependency. Students who engage more on Facebook may have less time studying, leading to poor academic performance. People need to be made aware of the issues associated with the misuse of Facebook.

## Introduction

Founded on 4 ^th^ February 2004 for communication limited to Harvard students, Facebook today is the most used social networking service (SNS) worldwide. There are 1.59 billion active Facebook users daily and 2.41 billion active Facebook users monthly as of June 2019 ^1^. The primary motives of Facebook use are to communicate, collaborate and share content ^2^. With such growing popularity and billions of users, there has been a concern of behavioral addiction to this SNS. Facebook addiction is said to occur when an individual spends an excessive amount of time on Facebook over the internet, disrupting one’s daily activities and social life ^3, 4^. If five hours or more is spent daily on Facebook then the person is said to be addicted to Facebook ^5^.

Excessive use of Facebook and other SNS is linked with health hazards including addiction and dependency. However, limited number of studies in this regard has made it a rarity in Nepalese context. The present study is therefore aimed to find out the level of Facebook addiction in a sample of Nepalese population and briefly discuss the issues associated with its unintended use.

## Methods

A descriptive cross-sectional study was conducted in Department of Forensic Medicine of Lumbini Medical College after obtaining ethical approval from the Institutional Review Committee vide the letter IRC-LMC 01-G/019.

The Bergen Facebook Addiction Scale (BFAS) is a questionnaire that comprises of six core features of addiction: salience, mood modification, tolerance, withdrawal, conflict, and relapse ^6^. Each of the six-core features consists of three questions, making a total of 18 questions. The final BFAS retained one question for each core element of addiction. Only the scores for questions 1, 5, 7, 11, 13 and 16 determine the level of Facebook addiction. Each question is scored on a 5-point Likert scale using anchors of 1: Very rarely and 5: Very often. Higher scores indicate greater Facebook addiction.

Participants scoring 4 (often) or 5 (very often) in four out of six questions were considered to be addicted to Facebook ^6, 7^. BASF has put forth two scoring schemes to determine Facebook addiction ^6^. As per a polythetic scoring scheme, Facebook addiction was determined by a liberal approach, where a score of 3 or more was observed in at least four of six items; whereas using a conservative approach, a score of 3 or above in all six items determined Facebook addiction by a monothetic scoring scheme ^6^.

Considering that there are around 1800 people in Lumbini Medical College including students, faculties and staffs, the sample size was calculated using the formula for finite population: n = N * X / (X+(N-1)); taking a confidence level of 95% (Z-score=1.96) and margin of error of 10%, the sample size was calculated to be 92.

The BFAS was typed into a Google Form and reviewed by all the authors for any mistakes which were then corrected. The link was then shared among the medical and nursing students, doctors, nurses and other health care staff working at Lumbini Medical College Teaching Hospital (LMCTH) through Facebook messenger, WhatsApp and Viber with a request to share the link among their friends or colleagues who were enrolled with LMCTH and were Nepalese citizens (convenient sampling). The first part of the questionnaire consisted of a statement where it was explained that no financial or material gifts will be provided for completing the questionnaire. The survey did not collect any identifying information of any of the participants and the responses were anonymous. The second part of the questionnaire was a section on consent where the participants had an option to choose whether they voluntarily consented to participate or didn’t consent. The third part of the questionnaire was accessible only to those participants who consented in the second part. The survey didn’t continue for the participants who didn’t consent and the incomplete form submitted. The link was made active on 27 July, 2019. On August 30, 2019 a total of 108 responses were received and the link was disabled from receiving further responses. There were five responses which were incomplete so those were excluded from the study. The obtained responses were downloaded as a Microsoft Excel spreadsheet, which was then exported into SPSS v16 for analysis. Descriptive statistics such as frequency, percentage, mean and standard deviation were used to determine demographic characteristics of the respondents and Facebook addiction (see *Underlying data*) ^8^.

## Results

The study sample consisted of 103 participants, of which 54 (52.42%) were males and 49 females (47.58%). There were 11 participants (10.68%) who had more than one Facebook account ( Table 1).

**Table 1.** Number of Facebook accounts of study participants.

Facebook accounts	Male N (%)	Female N (%)	Total N (%)
One	46 (44.66)	46 (44.66)	92 (89.32)
More than one	8 (7.76)	3 (2.92)	11 (10.68)
Total	54 (52.42)	49 (47.58)	103 (100)

The majority of participants (n=41, 39.8%) responded that during the last year they often spent a lot of time thinking about Facebook or planned use of Facebook. When asked if they felt an urge to use Facebook more and more, 35 participants (34.0%) felt they never felt such an urge. Only 16 (15.5%) responders used Facebook to forget about their personal problems during the last year. The present study showed that 23.3% of the responders (n=24) could not cut down their use of Facebook during the last year. When asked whether they became restless or troubled when prohibited from using Facebook, six participants (5.8%) responded that they often felt restless. In 21 participants (20.4%), their use of Facebook had a negative impact on their job/studies. The detailed responses of the participants are presented in Table 2 and Table 3.

**Table 2.** Items in the questionnaire and responses of the participants (n=103).

Items in the questionnaire	Responses in 5-point scale N (%)				
	1	2	3	4	5
How often during the last year have you spent a lot of time thinking about Facebook or planned use of Facebook?	10 (9.7)	10 (9.7)	42 (40.8)	28 (27.2)	13 (12.6)
How often in the last year have you felt an urge to use Facebook more and more?	11 (10.7)	24 (23.3)	39 (37.9)	22 (21.4)	7 (6.8)
How often during the last year have you used Facebook in order to forget about personal problems?	30 (29.1)	31 (30.1)	26 (25.2)	9 (8.7)	7 (6.8)
How often during the last year have you tried to cut down on the use of Facebook without success?	22 (21.4)	30 (29.1)	27 (26.2)	16 (15.5)	8 (7.8)
How often during the last year have you become restless or troubled if you have been prohibited from using Facebook?	42 (40.8)	37 (35.9)	18 (17.5)	3 (2.9)	3 (2.9)
How often during the last year have you used Facebook so much that it has had a negative impact on your job/studies?	26 (25.2)	29 (28.2)	27 (26.2)	20 (19.4)	1 (1.0)

**Table 3.** Items in the questionnaire and responses where the score is 4 or more (n=103).

Items in the questionnaire	Responses N (%)
How often during the last year have you spent a lot of time thinking about Facebook or planned use of Facebook?	41 (39.8)
How often in the last year have you felt an urge to use Facebook more and more?	29 (28.2)
How often during the last year have you used Facebook in order to forget about personal problems?	16 (15.5)
How often during the last year have you tried to cut down on the use of Facebook without success?	24 (23.3)
How often during the last year have you become restless or troubled if you have been prohibited from using Facebook?	6 (5.8)
How often during the last year have you used Facebook so much that it has had a negative impact on your job/studies?	21 (20.4)

There were ten participants (9.70%) who scored 4 or more in four questions. A 43-year-old male was among one of those seven participants who had multiple Facebook accounts and scored 5 in all the six questions. This was the maximum score one could get as per BFAS, which denotes this individual was severely addicted to Facebook use. As per the conservative approach there were only nine participants (8.73%) who were addicted to Facebook. When the liberal approach was used, there were 41 participants (39.80%) who were addicted to Facebook ( Table 4). 

**Table 4.** Facebook addiction of the study participants (n=103).

Facebook addiction	Gender N (%)		Total N (%)
	Male	Female	
Scored ≥4 in ≥4 items	4 (3.88)	6 (5.82)	10 (9.70)
Scored ≥3 in all 6 items (Conservative approach)	6 (5.82)	3 (2.91)	9 (8.73)
Scored ≥3 in ≥4 items (Liberal approach)	22 (21.36)	19 (18.44)	41 (39.80)

## Discussion

Although the popularity of Facebook is increasing daily, the fact that its use is prone to addiction cannot be denied ^9^. Many recent studies from around the world advocate the potential risk of addiction. There are various assessment tools and diagnostic criteria to investigate SNS addiction; however, BFAS is considered to have good psychometric properties ^10^. Facebook can be used for various purposes like instant messaging, video conferencing, gaming and shopping. Facebook is popular for its content sharing feature. The shared content may be news, personal blogs, pictures and videos, which can be educational, entertaining or explicit.

In a study conducted among postgraduate medical students in Southern India, it was observed that 26% of the participants were addicted to Facebook and 33% had the possibility of addiction ^11^. The study also concluded that loneliness influenced Facebook addiction.

A Nepalese study on Facebook use conducted among health science students of a private medical college showed that 98.2% were active Facebook users ^12^. The authors of the study concluded that 28.5% of the study participants were unable to reduce their time spent on Facebook and therefore were addicted. In contrast, the present study observed as little as 8.73% of participants addicted to Facebook use. Although the number may seem less, it still raises a concern over the use of SNS in Nepalese context. Excessive use of Facebook by students may lead to poor time management and less time studying, which would result in poor academic performance. Another reason for fewer participants with Facebook addiction in the present study might be the fact that the majority of the participants were health care professionals. There is restricted access to Facebook and other SNS sites in many hospitals. Medical schools including Lumbini Medical College, Palpa; Manipal Teaching Hospital, Pokhara and Nepal Medical College, Kathmandu have blocked access to Facebook from their internet servers.

A study conducted among 355 university students in the Philippines observed that only 15 were Facebook addicts ^7^. A study from the United States showed that nearly 96% of medical undergraduates regularly used Facebook for social life and academic pursuits ^13^. One of the studies conducted in Nepal stated Facebook to be a novel way of communication with members of health professional associations ^14^.

One of the underlying reasons for excessive use of Facebook may be narcissistic behavior in which the individual is frequently sharing content to gain interactions and positive feedback in an attempt to become popular and admired ^15^. The feeling of happiness tempts the individual to maintain and increase the level of happiness by engaging more on Facebook. Frequent use of Facebook can also lead to a person revealing his/her personal information like place of residence, job, family members etc. People should be made aware of the fact that this information can be used by individuals who take undue advantages or have ulterior motives. The increasing popularity of Facebook and other SNS sites is an indicator of people being more engaged in the online world. It has been observed that loneliness is one of the dominant predictors of social media addiction including Facebook ^16^. We are not far from receiving patients with a complaint of excessive use of SNS sites or the internet in Nepalese hospitals. The clinicians should be aware that treatment in case of Facebook addiction can never be complete abstinence as the internet has become an integral part of social culture. Treatment in such cases should be aimed at controlled use and identifying the underlying cause of compulsive overuse. 

The present study is not without limitations. The study participants were chosen through searching the researcher’s Facebook friend list, and there is a possible sampling bias in our study. As the study participants were requested to share the link among the students and employees of LMCTH, we could not calculate the number people approached and the response rate. The responses were collected anonymously and no identifying information of the responder was recorded; it therefore cannot be known if multiple responses form a same person occurred. Three different approaches were used in the present study that showed a varied addiction rate in the same population. BFAS is based upon use of Facebook, but it doesn’t specify the type of addiction is to technology or content. The convenient sampling, age, gender and occupational imbalance of the present study therefore cannot be generalized to the population of Nepal as a whole.

## Conclusions

Facebook addiction in the Nepalese context as per the present study was at least 8.73%, with a possibility a prevalence of addiction up to 39.80%. Complete abstinence from Facebook or any SNS is difficult to achieve as internet has become integral part of our lives; however, in case of excessive use patients can be advised to control their use. Considering the high popularity of Facebook and other SNS sites in Nepal, it is evident that there is a huge need for future research in this regard. 

## Data availability

### Underlying data

DRYAD: Cross-sectional study of Facebook addiction in a sample of Nepalese population. https://doi.org/10.5061/dryad.83bk3j9pv ^8^.

Data are available under the terms of the Creative Commons Zero "No rights reserved" data waiver (CC0 1.0 Public domain dedication).

## References

[1] Facebook Newsroom [Internet]. [cited 2019 Nov 18]. Reference Source

[2] Thurairaj S Hoon EP Roy SS: Reflections of students’ language usage in social networking sites: Making or marring academic English. *The Electronic Journal of e-Learning.* 2015;13(4):302–16. Reference Source

[3] Rana MS Ahmed O Hossain MA: Facebook addiction, self-esteem, and life satisfaction of Bangladeshi young people. *Dhaka Univ J Psych.* 2016;40:29–41. Reference Source

[4] Mekinc J Smailbegovic T Kokic A: Should we be concerned about children using the internet: pilot study. *Innov Issues Approaches Soc Sci.* 2013;6(2):6–20. 10.12959/issn.1855-0541.IIASS-2013-no2-art01

[5] Karaiskos D Tzavellas E Balta G: P02-232 - Social network addiction: a new clinical disorder? *Eur Psychiatry.* 2010;25(Supplement 1):855 10.1016/S0924-9338(10)70846-4

[6] Andreassen CS Torsheim T Brunborg GS: Development of a Facebook Addiction Scale. *Psychol Rep.* 2012;110(2):501–17. 10.2466/02.09.18.PR0.110.2.501-517 22662404

[7] Marcial DE: Are you a Facebook addict? Measuring Facebook addiction in the Philippine University. *Int Proc Econ Dev Res.* 2013;66:12–15. Reference Source

[8] Atreya A Nepal S Thapa P: Cross-sectional study of Facebook addiction in a sample of Nepalese population, v3, Dryad. Dataset2020 10.5061/dryad.83bk3j9pv PMC7726847 33489090

[9] Griffiths MD: Facebook addiction: concerns, criticism, and recommendations--a response to Andreassen and colleagues. *Psychol Rep.* 2012;110(2):518–20. 10.2466/01.07.18.PR0.110.2.518-520 22662405

[10] Kuss DJ Griffiths MD: Social Networking Sites and Addiction: Ten Lessons Learned. *Int J Environ Res Public Health.* 2017;14(3):311. 10.3390/ijerph14030311 28304359 PMC5369147

[11] Shettar M Karkal R Kakunje A: Facebook addiction and loneliness in the post-graduate students of a university in southern India. *Int J Soc Psychiatry.* 2017;63(4):325–329. 10.1177/0020764017705895 28504040

[12] Jha RK Shah DK Basnet S: Facebook use and its effects on the life of health science students in a private medical college of Nepal. *BMC Res Notes.* 2016;9:378. 10.1186/s13104-016-2186-0 27485717 PMC4970301

[13] Gafni R Deri M: Costs and benefits of facebook for undergraduate students. *Interdisciplinary Journal of Information, Knowledge, and Management.* 2012;7:45–61. Reference Source

[14] Chaudhary P Tuladhar H: Novel ways of improving communication with members of health professional associations. *Int J Gynaecol Obstet.* 2014;127 Suppl 1:S15–6. 10.1016/j.ijgo.2014.06.005 25000906

[15] Brailovskaia J Schillack H Margraf J: Facebook Addiction Disorder in Germany. *Cyberpsychol Behav Soc Netw.* 2018;21(7):450–456. 10.1089/cyber.2018.0140 29995531

[16] Biolcati R Mancini G Pupi V: Facebook Addiction: Onset Predictors. *J Clin Med.* 2018;7(6):118. 10.3390/jcm7060118 29882872 PMC6025609

