# Supplementary material for: Cross-sectional study of Facebook addiction in a sample of Nepalese population
Source: F1000Res. 2020 Dec 4;9:1237. Originally published 2020 Oct 14. [Version 2] doi: 10.12688/f1000research.26923.2 (PMC7726847; doi:10.12688/f1000research.26923.2)
Supplement: Peer review history

# Peer review: Reviewer response for version 2

**DOI:** 10.5256/f1000research.31035.r75720

**Published:** 2020-12-08

**Authors:** Abhishek Das

**Affiliations:** 1 Department of Forensic and State Medicine, Medical College, Kolkata, West Bengal, India

I have no further comments to make.

Is the work clearly and accurately presented and does it cite the current literature?

Yes

If applicable, is the statistical analysis and its interpretation appropriate?

Partly

Are all the source data underlying the results available to ensure full reproducibility?

Yes

Is the study design appropriate and is the work technically sound?

Partly

Are the conclusions drawn adequately supported by the results?

Yes

Are sufficient details of methods and analysis provided to allow replication by others?

Yes

Reviewer Expertise:

Forensic medicine, forensic psychiatry, medical education are my field of research.

I confirm that I have read this submission and believe that I have an appropriate level of expertise to confirm that it is of an acceptable scientific standard.

# Peer review: Reviewer response for version 2

**DOI:** 10.5256/f1000research.31035.r75721

**Published:** 2020-12-04

**Authors:** Kishor Adhikari

**Affiliations:** 1 Department of Community Medicine and Public Health, Chitwan Medical College, Bharatpur, Nepal; 2 Himalayan Environment and Public Health Network (HEPHAN), Bharatpur, Nepal

Now it seems good. I am okay with the modifications made along with the clarifications made by the authors.

Is the work clearly and accurately presented and does it cite the current literature?

Yes

If applicable, is the statistical analysis and its interpretation appropriate?

Partly

Are all the source data underlying the results available to ensure full reproducibility?

Yes

Is the study design appropriate and is the work technically sound?

Yes

Are the conclusions drawn adequately supported by the results?

Partly

Are sufficient details of methods and analysis provided to allow replication by others?

Partly

Reviewer Expertise:

NA

I confirm that I have read this submission and believe that I have an appropriate level of expertise to confirm that it is of an acceptable scientific standard.

# Peer review: Reviewer response for version 1

**DOI:** 10.5256/f1000research.29737.r74539

**Published:** 2020-11-20

**Authors:** Abhishek Das

**Affiliations:** 1 Department of Forensic and State Medicine, Medical College, Kolkata, West Bengal, India

The cross sectional study done is presented well with originality.

 The Abstract **reflects the major aspects of the main paper. However, the **different approaches** mentioned here needs a little elaboration. Inclusion of the keyword " **Facebook**" would be beneficial.

 The Introduction properly reflects the background of study, but needs to highlight more on existing gap in literature. The **level of addiction** should be clarified clearly.

 The Methods section is well written and can be replicated later by others. However the **convenience sampling** could have been improved by choosing the portion of general Nepali population attending hospital apart from only including those attached to LMCTH. Exactly how many participants approached (google form sent) was not mentioned clearly, so the **response rate** to BFAS in this study could not be calculated. The response to the SIX core features of the addiction was not mentioned in detail in Results section.

 Two wrong representations of data noted:

1. 'BAFS' is to be replaced by **BFAS **in the first line of last paragraph of *methods *section.
2. In the description of Tables 2 & 3 on "who could not cut down their use of Facebook during the last year" the result should be n=24 without the % sign.

Overall, this is a well formulated and executed study with authentic results.

Is the work clearly and accurately presented and does it cite the current literature?

Yes

If applicable, is the statistical analysis and its interpretation appropriate?

Partly

Are all the source data underlying the results available to ensure full reproducibility?

Yes

Is the study design appropriate and is the work technically sound?

Partly

Are the conclusions drawn adequately supported by the results?

Yes

Are sufficient details of methods and analysis provided to allow replication by others?

Yes

Reviewer Expertise:

Forensic medicine, forensic psychiatry, medical education are my field of research.

I confirm that I have read this submission and believe that I have an appropriate level of expertise to confirm that it is of an acceptable scientific standard, however I have significant reservations, as outlined above.

# Response

**Published:** 2020-11-25

**Authors:** Alok Atreya

**Affiliations:** Lumbini Medical College, Nepal

We would like to thank the learned reviewer for the detailed review and positive comments on our manuscript. Based upon the comments the following changes are made:

 Comment 1: *The cross-sectional study done is presented well with originality.*

**Response:** Thank you for the comment.

 Comment 2: *The Abstract reflects the major aspects of the main paper. However, the different approaches mentioned here needs a little elaboration.*

**Response: **The different approaches that were used for e.g. polythetic and monothetic scoring scheme are detailed in the methods and results section so it was removed from here to avoid confusion. The sentence “When different approaches were applied it was observed that 8.73% (n=9) to 39.80% (n=41) were addicted to Facebook.” is reframed to “It was observed that 8.73% (n=9) to 39.80% (n=41) were addicted to Facebook.”

 Comment 3: *Inclusion of the keyword "Facebook" would be beneficial.*

**Response:** Facebook is added as a keyword.

 Comment 4: T *he Introduction properly reflects the background of study, but needs to highlight more on existing gap in literature. The level of addiction should be clarified clearly.*

**Response: **The last paragraph is elaborated as:

 “Excessive use of Facebook and other SNS is linked with health hazards including addiction and dependency. However, limited number of studies in this regard has made it a rarity in Nepalese context. The present study is therefore aimed to find out the level of Facebook addiction in a sample of Nepalese population and briefly discuss the issues associated with its unintended use.”

 Comment 5: *The Methods section is well written and can be replicated later by others. However the convenience sampling could have been improved by choosing the portion of general Nepali population attending hospital apart from only including those attached to LMCTH.*

**Response: **We are grateful for the suggestion and will keep it in mind for future works. The study population for the present study are students (medical and nursing) and employees of Lumbini Medical College, Palpa. Although, the Nepalese students studying in the college are from different parts of the country and so are the employees; we have used the term ‘sample of Nepalese population’.

 Comment 6: *Exactly how many participants approached (google form sent) was not mentioned clearly, so the response rate to BFAS in this study could not be calculated.*

**Response: **This was one of the limitations of the study. We have added the following sentence as limitation: “As the study participants were requested to share the link among the students and employees of LMCTH, we could not calculate the number people approached and the response rate.”

 Comment 7: *The response to the SIX core features of the addiction was not mentioned in detail in Results section.*

**Response:** Table 2 and Table 3 detail the responses on the six core features of addiction.

 Comment 8: *Two wrong representations of data noted:*

*'BAFS' is to be replaced by BFAS in the first line of last paragraph of methods section.*

*In the description of Tables 2 & 3 on "who could not cut down their use of Facebook during the last year" the result should be n=24 without the % sign.*

**Response: **Corrected.

 Comment 9: *Overall, this is a well formulated and executed study with authentic results.*

**Response:** Thank you.

# Peer review: Reviewer response for version 1

**DOI:** 10.5256/f1000research.29737.r73468

**Published:** 2020-11-03

**Authors:** Kishor Adhikari

**Affiliations:** 1 Department of Community Medicine and Public Health, Chitwan Medical College, Bharatpur, Nepal; 2 Himalayan Environment and Public Health Network (HEPHAN), Bharatpur, Nepal

- Title: The authors have taken samples from only one medical college of Nepal, so, it may not look correct to state as "Nepalese population".
- Method: There is no sampling technique as "Convenient random sampling". It is better to keep as convenient sampling. 
- Conclusion: Don't keep many things which are not the findings of your research. 

Is the work clearly and accurately presented and does it cite the current literature?

Yes

If applicable, is the statistical analysis and its interpretation appropriate?

Partly

Are all the source data underlying the results available to ensure full reproducibility?

Yes

Is the study design appropriate and is the work technically sound?

Yes

Are the conclusions drawn adequately supported by the results?

Partly

Are sufficient details of methods and analysis provided to allow replication by others?

Partly

Reviewer Expertise:

Non communicable disease risk factors, health system

I confirm that I have read this submission and believe that I have an appropriate level of expertise to confirm that it is of an acceptable scientific standard, however I have significant reservations, as outlined above.

## References

[1] : Facebook use and its effects on the life of health science students in a private medical college of Nepal. *BMC Res Notes*.2016;9: 10.1186/s13104-016-2186-0 378 10.1186/s13104-016-2186-0 27485717PMC4970301

# Response

**Published:** 2020-11-25

**Authors:** Alok Atreya

**Affiliations:** Lumbini Medical College, Nepal

We would like to thank the learned reviewer for the detailed review and positive comments on our manuscript. Based upon the comments the following changes are made:

 Comment 1 Title: *The authors have taken samples from only one medical college of Nepal, so, it may not look correct to state as "Nepalese population".*

**Response:** The study population for the present study are students (medical and nursing) and employees of Lumbini Medical College, Palpa. The Nepalese students studying in the college are from different parts of the country and so are the employees. However, the whole Nepalese population cannot be represented by this study we have used the term ‘sample of Nepalese population’ in the title.

 Comment 2 Method: *There is no sampling technique as "Convenient random sampling". It is better to keep as convenient sampling.*

**Response: **Changed as instructed.

 Comment 3 Conclusion: *Don't keep many things which are not the findings of your research.*

**Response:** Conclusion is shortened and only the relevant findings are included.
